# Aerobic Exercise during Pregnancy and Presence of Fetal-Maternal Heart Rate Synchronization

**DOI:** 10.1371/journal.pone.0106036

**Published:** 2014-08-27

**Authors:** Peter Van Leeuwen, Kathleen M. Gustafson, Dirk Cysarz, Daniel Geue, Linda E. May, Dietrich Grönemeyer

**Affiliations:** 1 Grönemeyer Institute of Microtherapy, University of Witten/Herdecke, Bochum, Germany; 2 Hoglund Brain Imaging Center and Department of Neurology, University of Kansas, Kansas City, Kansas, United States of America; 3 Integrated Curriculum for Anthroposophic Medicine, University of Witten/Herdecke, Herdecke, Germany; 4 Research and Development, VISUS Technology Transfer GmbH, Bochum, Germany; 5 Department of Foundational Sciences and Research, East Carolina University, Greenville, North Carolina, United States of America; University of Adelaide, Australia

## Abstract

It has been shown that short-term direct interaction between maternal and fetal heart rates may take place and that this interaction is affected by the rate of maternal respiration. The aim of this study was to determine the effect of maternal aerobic exercise during pregnancy on the occurrence of fetal-maternal heart rate synchronization.

**Methods:**

In 40 pregnant women at the 36^th^ week of gestation, 21 of whom exercised regularly, we acquired 18 min. RR interval time series obtained simultaneously in the mothers and their fetuses from magnetocardiographic recordings. The time series of the two groups were examined with respect to their heart rate variability, the maternal respiratory rate and the presence of synchronization epochs as determined on the basis of synchrograms. Surrogate data were used to assess whether the occurrence of synchronization was due to chance.

**Results:**

In the original data, we found synchronization occurred less often in pregnancies in which the mothers had exercised regularly. These subjects also displayed higher combined fetal-maternal heart rate variability and lower maternal respiratory rates. Analysis of the surrogate data showed shorter epochs of synchronization and a lack of the phase coordination found between maternal and fetal beat timing in the original data.

**Conclusion:**

The results suggest that fetal-maternal heart rate coupling is present but generally weak. Maternal exercise has a damping effect on its occurrence, most likely due to an increase in beat-to-beat differences, higher vagal tone and slower breathing rates.

## Introduction

Normal prenatal development involves changes in fetal heart rate reflected in a gradual slowing of rates and accompanied by increases in heart rate variability (HRV). These changes result from the overall prenatal development of the autonomic nervous system, the changing internal physiological demands and the increasing interaction with the intrauterine environment. For example, as pregnancy progresses the increased occurrence of fetal waking and sleeping states, gross or fine motor activity, eye movements and breathing movements have all been associated with changes in fetal heart rate and HRV [1] [2] [3]. Also, external stimuli such as music or speech lead to vibroacoustic stimulation which can affect fetal HRV [4] [5]. Some of the external conditions affecting the fetus originate in the maternal state. It has been shown that maternal hypoxia [6], hypothermia [7], stress [8], relaxation [9] or emotive state [10] can influence fetal HRV.

Maternal activity in the form of exercise will also elicit fetal heart rate response. For instance, fetal heart rate increases during maternal exercise and, after exercise cessation, drops back to baseline rates [11]. More recently it has been shown by some of us that regular aerobic exercise during pregnancy affects overall fetal heart rate [12]. The fetuses of women who exercised during pregnancy had lower heart rates and increased HRV relative to fetuses that were not exposed to maternal exercise. This suggests a supportive effect on the development of the fetal autonomic nervous system in the exercise group.

Other work that some of us have done suggests that direct interaction between maternal and fetal heart rates may take place [13]. This interaction manifests itself as recurring short episodes of heart rate synchronization between mother and child of around 15 s duration. As the occurrence of synchronization varied substantially in subjects and data sets, we attempted to identify possible physiological conditions which may hinder or promote this interaction. Further work showed that fetal-maternal heart rate synchronization occurred significantly more often under conditions of relatively fast maternal breathing and less at slow breathing rates [14] [15]. It was postulated that the enhancement (or suppression) of maternal HRV associated with high (or low) respiration rates may create conditions which facilitate (or impede) the fetal cardiac system to couple its timing to that of the maternal system. In order to further investigate this, the aim of this study was to examine the occurrence of fetal-maternal heart rate synchronization in pregnancies in which the mothers were either exercising regularly or were sedentary. The hypothesis was that the differences in heart rate and HRV in both the mothers and their fetuses resulting from exercising/not exercising will lead to differences in the heart rate synchronization characteristics between the two groups. Such differences could help further elucidate the mechanisms involved in fetal-maternal heart rate interaction.

## Methods

### Subject Population

This was a retrospective analysis of fetal-maternal magnetocardiograms (MCGs) recorded from women who were enrolled in a study that was designed to measure the effect of maternal physical activity on the longitudinal development of fetal cardiac autonomic control. Methods and results of the study are previously published [12] [16] but will be presented briefly here. The study was approved by the Kansas City University of Medicine and Biosciences and University of Kansas Medical Center Institutional Review Boards, following the tenets of the Declaration of Helsinki. Recruitment was limited to low-risk, 20–35 year-old women carrying singleton pregnancies. All the women agreed to the data acquisition and for their records to be used in the study. They provided written informed consent stating as much for themselves and their unborn children prior to participation. All data were anonymized and de-identified prior to analysis.

Women were categorized into the Exercise group based on their response to the Modifiable Physical Activity Questionnaire (MPAQ) [17]. Women who engaged in moderate to vigorous aerobic exercise for a minimum of 30 minutes, 3 times per week throughout pregnancy were assigned to the Exercise group. Those who did not meet the above criterion were assigned to the Control group.

### Data Acquisition

Data were acquired using an investigational 83-channel dedicated fetal biomagnetometer (CTF Systems, Inc., subsidiary of VSM MedTech Ltd., Vancouver, Canada). A continuous, 18 minute simultaneous fetal-maternal MCG was recorded for each subject. Women were tested in a magnetically shielded room to eliminate the influence of electromagnetic artifacts. The pregnant subjects were comfortably seated in a slightly reclined position in front of the biomagnetometer and monitored via video camera and microphone. The data were acquired using a 300 Hz sampling rate and recording filter of 0–75 Hz.

### Data Processing

Data were digitally filtered between 1 and 40 Hz offline (bidirectional fourth-order Butterworth filter) then subjected to independent component analysis (ICA) using EEGLAB toolbox (version 4.311). ICA is a blind-source separation technique used to segregate the contributions from multivariate and spatially distinct electrophysiological sources into individual components (e.g., maternal heart, fetal heart, fetal diaphragm). Maternal and fetal MCG components were identified and R-peaks marked using software developed by our team [12]. We previously established through longitudinal measures obtained at 28, 32 and 36 weeks gestational age that group differences emerged at 36 weeks. Therefore, analysis for fetal-maternal synchronization characteristics was limited to data collected at 36 weeks. Datasets with excessive artifacts (ectopic beats, preventricular or preatrial contractions) were excluded from the analysis. Forty datasets were used for this retrospective analysis (Control: n = 19; Exercise: n = 21).

**Figure 1:**
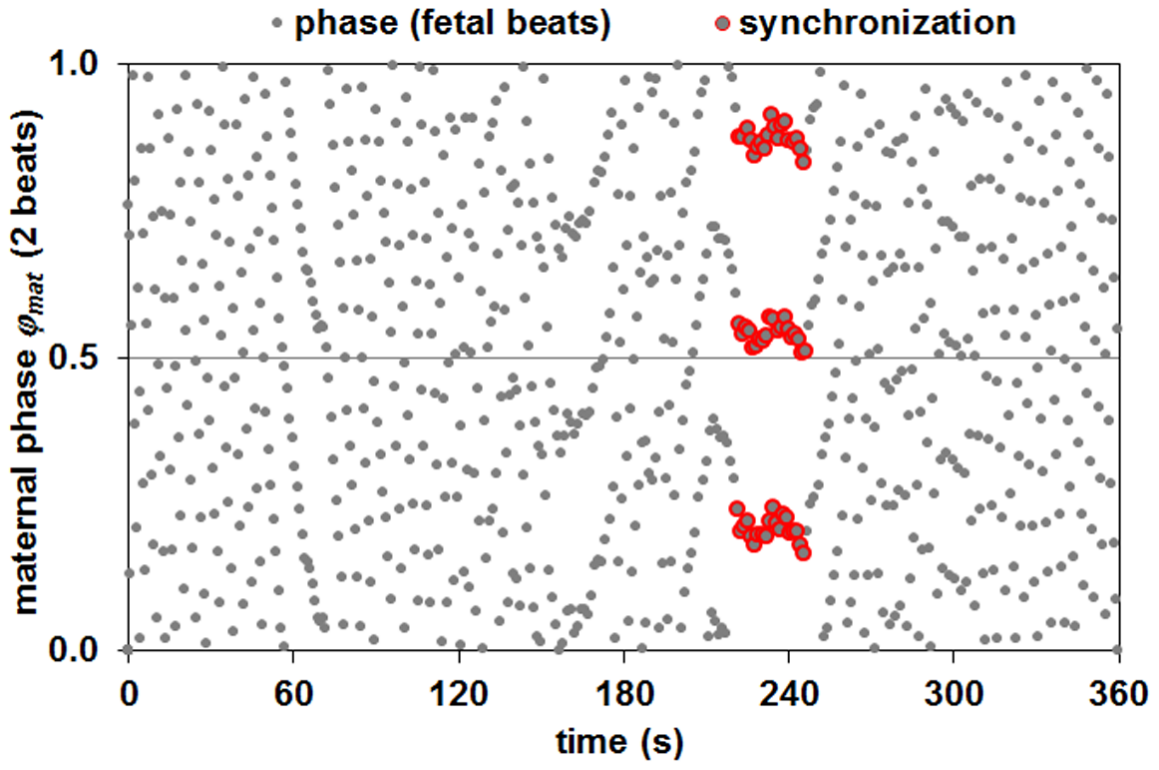
Six minute synchrogram showing a 25 s episode of synchronization starting at 221 s with three fetal beats occurring within two maternal beats at constant phase values (*n*:*m* = 3:2).

Each bivariate data set consisted of the fetal and maternal R peak times. From these data sets, timings of the fetal R peaks relative to maternal R peaks were determined. Synchrograms were constructed by plotting the phases of the fetal beats with respect to *m* maternal RR interval cycles (1≤ *m* ≤4) over time [13]. As in our previous work, we normalized the timing of these phases *φ_mat_* to values between 0 and 1 (corresponding to 0 and 2π). Episodes in which the phase timing *φ_mat_* remained constant for at least 10 s were considered to indicate fetal-maternal heart rate synchronization. In such episodes, the fetal beats within the maternal RR interval cycle were visible as *n* parallel horizontal groupings of data points (see Fig. 1). The identification of these groupings as synchronization epochs (SE) was performed using an algorithm developed by Toledo et al. [18], whereby the threshold values were dependent on the number of maternal cycles (threshold = 0.03*m) [13]. Based on the ranges of maternal and fetal heart rates, *n* was set 2≤ *n* ≤9 and we examined the non-redundant *n*:*m* combinations 2:1, 3:2, 4:3, 5:3, 7:3, 5:4, 7:4, 9:4.

### Data analysis

In each 18 min. mother-fetus data set, we determined maternal and fetal HRV, the maternal respiratory rate and the number of SE found was noted. Each SE was characterized by its duration, *n*:*m* ratio, maternal and fetal RR interval duration and HRV, their bivariate variability *Δq*, the maternal phase *φ_mat_* and maternal respiratory rate. The occurrence and characteristics of the SE data were examined in the maternal-fetal pairs of the two groups.

With respect to the calculation of *φ_mat_* we took the following into consideration. We postulate that, in the case of physiological interaction, there will be a more or less fixed offset of the fetal beat timing with respect to the timing of the initial maternal beat, i.e. the *φ_mat_* values will be similar. For instance, for any identified SE with a 2:1 combination, there will be two values of *φ_mat_*: *φ_mat1_* and *φ_mat2_*. For those SE which result from synchronization, the *φ_mat1_* values (and *φ_mat2_* values, respectively) will be similar. The *φ_mat1_* and *φ_mat2_* values of those SE which do not reflect physiological interaction but result from chance will be arbitrary and can take on any value between 0 and 1. Importantly, for *n*:*m* combinations with more than one beat (*m* >1), the value of *φ_mat1_* for synchronized SE will take on *m* different values depending on which of the *m* beats is first identified by the Toledo algorithm [18]. E.g. the offset *φ_mat1_* in a 3:2 SE with phase preference may take on one of two values, depending on whether the first of the 2 maternal RR interval identified (*m* = 2) contains 1 or 2 of the fetal beats (*n* = 3) (see also Fig. 1). In the analysis of possible phase preferences in the SE, we took this into account by adjusting the *φ_mat_* values as follows: *φ_mat-adj_* = *φ_mat_* mod (1/*m*). *φ_mat-adj_* was calculated only for the SE found in *n*:*m* combinations with at least 10% of all SE found (i.e. 3:2, 4:3, 5:3, 7:4, see Results).

With respect to the maternal respiratory rate, no direct measurement of the mothers’ breathing was performed during the data acquisition. Therefore the instantaneous maternal respiratory rate was estimated on the basis of ECG derived respiration (EDR) [19]. EDR evaluates and quantifies the respiration induced variations of the R-wave amplitude in the ECG. This approach was implemented here on the MCG signals of the mothers and permitted an assignment of respiratory rates on a beat-to-beat basis. The respiratory rate of a complete 18 min. data set or within the single SE was characterized by the median of the beat-to-beat rates.

Due to natural variations in both maternal and fetal heart rates, spurious transient episodes of constant phase timing of fetal beats may occur without any basis in physiological processes. In order to distinguish between such chance coordination and real synchronization, we constructed surrogate data sets in which the timing relationships between the fetal and maternal heart beats were destroyed. This was done by creating so-called twin surrogate data sets of the maternal data based on the recurrence matrix [20]. Details of the surrogate data approach can be found in [21] and the details of the calculation of the twin surrogates can be found in [14]. For the calculation of the twin surrogates we set the embedding dimension to 3 and used a delay of 1. In order to achieve stable statistical results we produced 10 surrogate data sets for each original set. The surrogate data sets consisting of the shuffled maternal R times and the original fetal R times were processed and analyzed in the same fashion as the original data and the results were compared to those of the original data. If no differences between the surrogate and original data become apparent, then the results in the original data may be due simply to chance and have no physiological significance.

### Statistics

Values are given as mean ± standard deviation (SD) or as median with the 90% confidence intervals (CI), as appropriate. In the presentation of the results in the text, Tables and Figures, it was at times expedient to divide the number of surrogate SE by ten in order to present the results of the original and surrogate data in the same order of magnitude. The analyses were nonetheless performed on all data.

HRV was quantified in the time domain for both the maternal and fetal RR time series using the SD of all normal-to-normal beats (SDNN) as a measure for overall variance and the root mean square of successive differences (RMSSD) of the normal-to-normal beats as a measure for short term variability. The bivariate variability *Δq* was calculated on the basis of the means and SDs of the fetal and maternal RR intervals as *Δq* = sqrt[(*RR_mat_*/*RR_fet_* ^2^ × *SD_fet_*)^2^+(1/*RR_fet_* × *SD_mat_*)^2^], where *RR* = mean RR interval, *SD* = SDNN and the subscripts *mat* and *fet* stand for the corresponding maternal and fetal data [22]. *Δq* has no dimension.

The order of the *n*:*m* combinations used to display the data is: 5:4, 4:3, 3:2, 5:3, 7:4, 2:1, 9:4, 7:3. This corresponds to a decrease in the ratio of fetal to maternal beats (0.80, 0.75, 0.67, 0.60, 0.57, 0.50, 0.44, 0.43, respectively) and indicates an order starting with similar fetal and maternal heart rates to fetal heart rates much faster than the maternal. The differences in distributions of the number of SE over the *n*:*m* combinations were tested using Pearson’s Chi^2^ test. The Mann-Whitney U test was used to examine differences between the Control and Exercise subjects with respect in the number of SE, SE duration, the maternal and fetal HRV measures and maternal respiratory rates. This test was also used to examine the corresponding group differences between the original and surrogate data. The differences in the duration of the SE in the different *n*:*m* combinations was tested using the Kruskal-Wallis test. The dependency of the number of SE per data set on HRV measures as well as on respiratory rate was quantified on the basis of linear regression. The strength of the relationships was given using the correlation coefficient *r* and the amount of variance explained by the coefficient of determination *r* ^2^. The frequency distribution of the *φ_mat-adj_* values was examined for each *n*:*m* combination, both for the original and surrogate data, in histograms with a 5*n bin size. The distribution of the phases in the histograms was tested by fitting partial Fourier series to the data using the general linear least-squares model and the significance of the fit was estimated by the zero-amplitude test (F-statistic) [23]. Statistical significance was considered achieved at the level *p*<0.05 and all values were interpreted in an explorative manner.

## Results

### Overall heart rate and HRV in the subjects

RR interval duration in the data sets of the mothers varied considerably, ranging from 573 ms to 1028 ms (heart rate range: 58–105 bpm). The values did not differ significantly between those who had not performed exercise and those who had. Overall variance (SDNN) was also similar but short term variability (RMSSD) tended to be higher in the Exercise group (Table 1). In the fetuses, RR interval duration ranged from 373–518 ms (heart rate range: 116–161 bpm) and tended to be shorter in the fetuses whose mothers had not performed exercise. Both overall variance and short term variability were significantly greater in the Exercise group (Table 1). The combined fetal-maternal variance, quantified on the basis of *Δq*, showed that the data sets of Control group had significantly lower variance than those of the Exercise group: 0.134±0.024 vs. 0.172±0.041, *p* = 0.004. We did not find significant differences between the maternal HRV measures in the original 18 min. data sets and those in the surrogate data sets (Table 1).

**Table 1:** Fetal and maternal mean RR interval duration, standard deviation (SDNN) and root mean square of successive differences (RMSSD) found in the subjects’ 18 min. data sets as well as in their synchronization epochs (SE).

			fetal			maternal	
		Control	Exercise		Control	Exercise	
		n = 19	n = 21	*p*-value	n = 19	n = 21	*p*-value
RR interval (ms)							
18 min.	original	427±33	445±30	0.074	674±62	718±116	0.226
18 min.	surrogate	n.a.	n.a.		676±63	719±115	0.258
SE	original	420±35	430±34	0.294	646±59	691±122	0.294
SE	surrogate	430±33	442±28	0.294	672±65	715±118	0.187
SDNN (ms)							
18 min.	original	21.4±6.2	29.3±9.6	**0.012**	44.9±12.5	57.6±22.2	0.138
18 min.	surrogate	n.a.	n.a.		44.4±12.8	56.6±22.0	0.124
SE	original	5.3±1.5	6.2±1.5	0.054	13.5±4.1	19.1±12.9	0.093
SE	surrogate	6.6±1.6	8.1±2.2	**0.044**	17.0±4.9	24.2±14.0	0.057
RMSSD (ms)							
18 min.	original	6.2±2.1	8.6±3.8	**0.015**	20.8±9.1	35.2±26.9	0.074
18 min.	surrogate	n.a.	n.a.		21.1±9.1	35.8±27.9	0.074
SE	original	4.1±1.1	4.5±1.3	0.258	12.3±6.0	20.3±19.7	0.083
SE	surrogate	4.8±1.5	5.7±1.6	0.054	16.2±7.8	25.3±21.1	0.099

All values are given for the original data as well as the surrogate data, except for the 18 min. fetal data sets (only the maternal data sets were shuffled, see text). The values are compared between the Control and Exercise groups. *p*-values <0.05 in **bold type**.

n.a., not applicable.

### Number of synchronization epochs (SE)

In the 40 data sets examined, we found a total of 676 SE in the original data, considerably more in the Control than in the Exercise group (403 vs. 273). Most of the SE (88%) were in the *n*:*m* combinations 4:3, 3:2, 5:3 and 7:4 (Table 2). The distributions over the *n*:*m* combinations differed significantly overall between the Control and Exercise groups (*p*<0.001) and in particular for *n*:*m* = 4:3, 3:2, 2:1 and 9:4.

**Table 2:** Number of synchronization epochs in the original and surrogate data sets, overall and in the various *n*:*m* combinations (corresponding percent values in parentheses).

	all				*n*:*m*				
		5:4	4:3	3:2	5:3	7:4	2:1	9:4	7:3
original									
all	676	26 (4)	102 (15)	233 (34)	181 (27)	81 (12)	23 (3)	21 (3)	9 (1)
Control	403	14 (3)	50 (12)	151 (37)	119 (30)	52 (13)	16 (4)	1 (0)	0 (0)
Exercise*	273	12 (4)	52 (19)	82 (30)	62 (23)	29 (11)	7 (3)	20 (7)	9 (3)
surrogate									
all	687.8	20.8 (3)	101.7 (15)	250.9 (36)	167.3 (24)	92.4 (13)	28.7 (4)	16 (2)	9.7 (1)
Control	402.2	8.4 (2)	45.9 (11)	157 (39)	111.3 (28)	61.6 (15)	16.7 (4)	0.8 (0)	0.5 (0)
Exercise*	285.6	12.4 (4)	55.8 (20)	93.9 (33)	56 (20)	30.8 (11)	12 (4)	15.2 (5)	9.2 (3)

The surrogate absolute values have been divided by ten for comparison with the original values.

*distribution over *n*:*m* significantly different from Control (*p*<0.001).

In the 10 sets of surrogate data, on average 687.8 SE/set were found, a few more than in the original data. The ratio of SE found in the Control and Exercise groups was similar to that in the original data (402.2 vs. 285.6). The distribution over the *n*:*m* combinations was not different between the original and surrogate data (*p* = 0.50, Table 2). Within the surrogate data, the distributions over the *n*:*m* combinations differed significantly overall between the Control and Exercise groups (*p*<0.001) and were similar to those in the original data.

In the original data the number of SE per subject was 16.9±7.4. There were significantly more SE in the Control group than in the Exercise group (21.2±5.9 vs. 13.0±6.5, *p*<0.001). Compared to the original data, the number of SE per subject in the surrogate data was not significantly different (17.2±7.2, *p* = 0.84). As in the original data, the number was higher in the surrogate Control group than in the surrogate Exercise group (21.2±5.4 vs. 13.6±6.9, *p* = 0.001).

**Figure 2:**
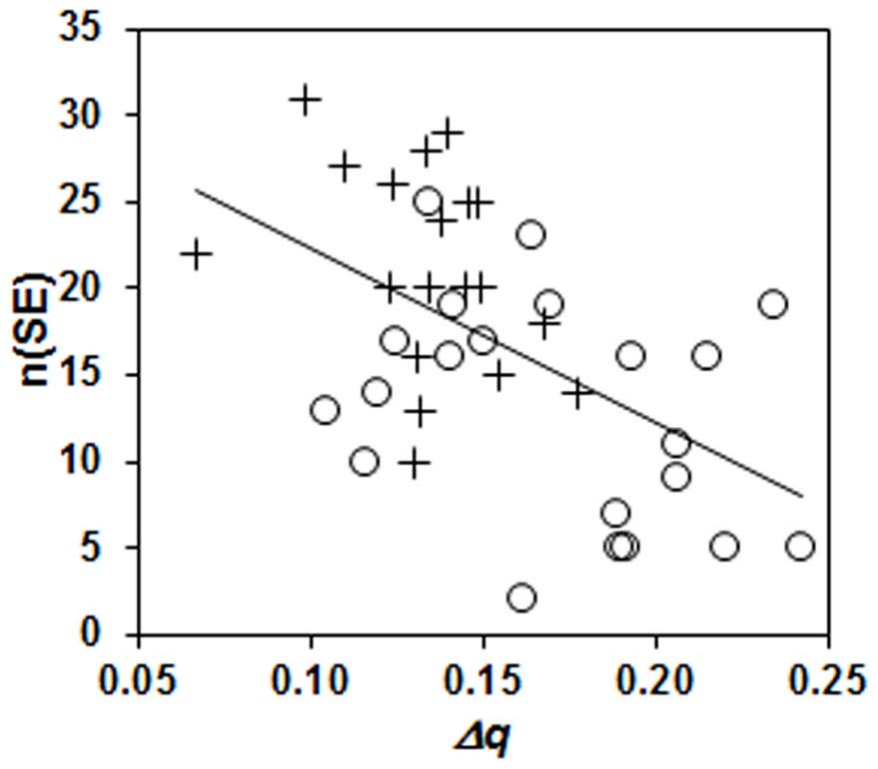
Relationship between the overall fetal-maternal variance (*Δq*) and the number of synchronization epochs (SE) found per data set. Data points are shown separately for the Control (+) and Exercise (O) data, the regression line shown is calculated over all data points.

The number of SE found in a single 18 min. data set was significantly dependent on both fetal and maternal HRV as well as their combined variance (Table 3). By way of example, the relationship between *Δq* and n(SE) is shown in Fig. 2.

**Table 3:** Dependency of the number of SE found on fetal and maternal heart rate variability.

	slope			
	n(SE)/ms*	*p*_slope_	*r*	*r^2^*
fetal RR interval	−0.05	0.158	−0.227	0.052
fetal SDNN	−0.38	**0.003**	−0.461	0.213
fetal RMSSD	−0.98	**0.005**	−0.438	0.192
maternal RR interval	−0.02	0.105	−0.260	0.068
maternal SDNN	−0.14	**0.027**	−0.349	0.122
maternal RMSSD	−0.18	**0.001**	−0.509	0.259
*Δq*	−101	**<0.001**	−0.526	0.277

SDNN: standard deviation, RMSSD: root mean square of successive differences, *Δq*: combined fetal-maternal variance, *r*: coefficient of correlation, *r^2^*: coefficient of determination. *p*_slope_ values <0.05 in **bold type**.

*except *Δq*.

### Duration of synchronization epochs (SE)

Valid SE were defined as having a duration ≥10 s. The mean duration of the SE in the original data was 16.4±8.8 s (90% CI: 10–31 s). No differences were found between the Control and Exercise groups nor in their respective distributions over *n*:*m* combinations. Due to the higher number of SE per subject in the Control group, the total SE time per subject was significantly longer in this group compared to the Exercise group (366±141 s vs. 215±121 s, *p* = 0.003).

SE duration was shorter in the surrogate data (15.8±7.3, 90% CI: 10–30 s; *p* = 0.035). In contrast to the original data, the durations of the surrogate Exercise SE were significantly shorter than those of the Control (13.7±1.1 vs. 12.7±1.1 s, *p*<0.015). Also the distributions over the *n*:*m* combinations was not uniform (*p*<0.001). But, as in the original data, the total duration of SE episodes per subject was longer in the Control group (348±128 s vs. 202±111 s, *p* = 0.001).

### Heart rate variability in the SE

For each SE that was identified, we calculated the mean RR interval, SDNN and RMSSD for both the mother’s and the fetus’ data. Comparing the subjects’ values between the Control and Exercise groups in the original data, we found no differences with respect to RR interval (Table 1). There was a tendency for slightly higher fetal and maternal SDNN and maternal RMSSD in the exercise group but no statistically significant differences could be found. Examination of the bivariate fetal-maternal variability also showed only a statistical trend in the difference between the *Δq* values of the Control and Exercise subjects: 0.043±0.011 vs. 0.054±0.030, *p* = 0.078. The results in the surrogate data were similar, whereby a significant difference was found for fetal RMSSD (Table 1) as well as for *Δq* (0.050±0.012 vs. 0.067±0.035, *p* = 0.034).

### Phase timing *φ_mat-adj_*

**Figure 3:**
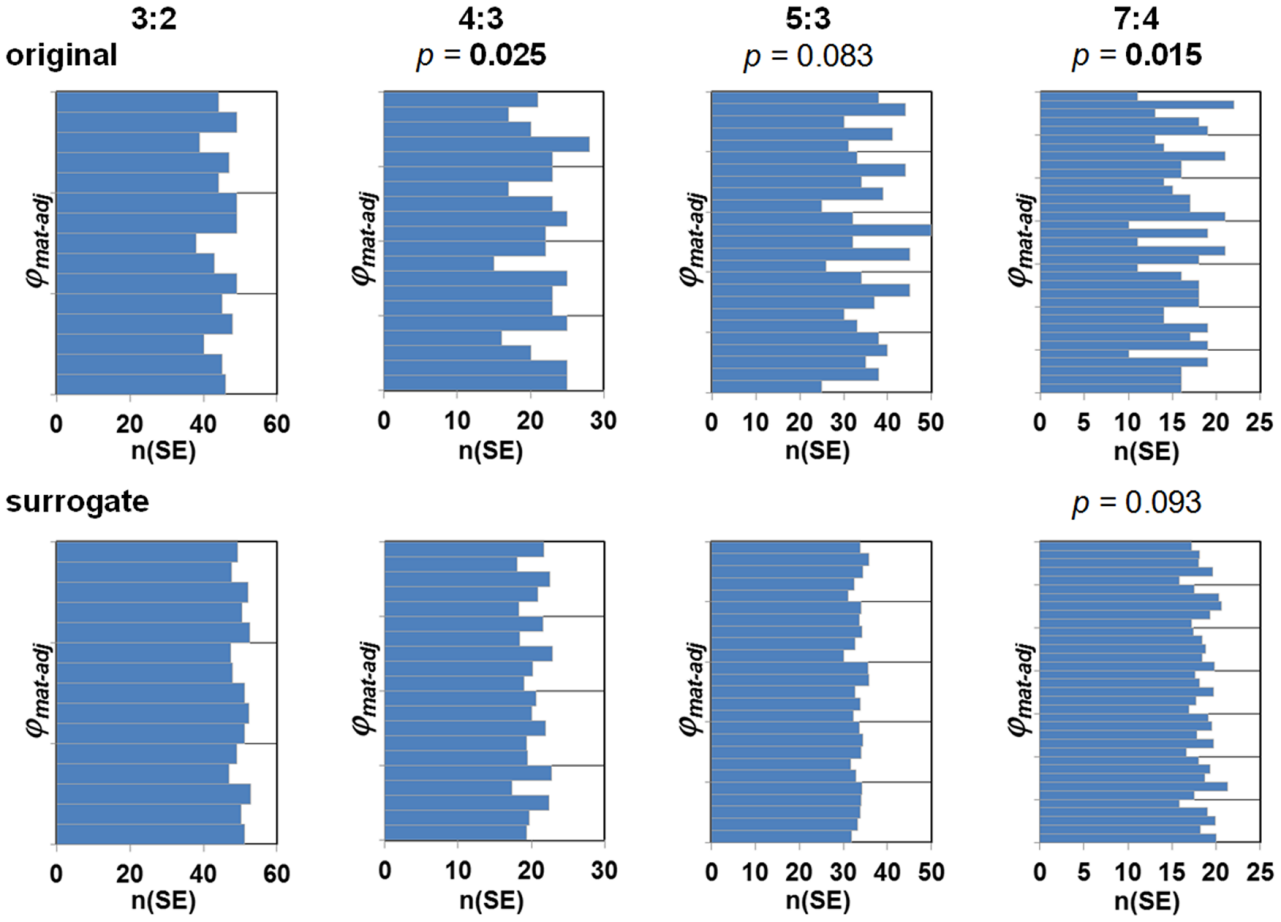
Distribution of the synchronization epochs (SE) over the maternal beat phase *φ_mat-adj_* in the original (top row) and surrogate data (bottom row) with respect to the *n*:*m* combinations 3:2, 4:3, 5:3 and 7:4. For the analysis, there were 10 surrogate data sets for each original; the histograms show the number of surrogate SE divided by 10 for comparability. *p*-values <0.10 for non-uniform distributions are given above the respective histograms, *p*-values <0.05 in **bold type**.

The distributions of the SE phases *φ_mat-adj_* was examined for the *n*:*m* combinations 4:3, 3:2, 5:3 and 7:4. Histograms were constructed for each *n*:*m* combination with 5*n bins. In the original data there was a tendency to non-uniform distributions with roughly *n* peaks and troughs (Fig. 3). The fit to a sinus function was statistically significant for 4:3 (*p* = 0.025) and 7:4 (*p* = 0.015) and with a trend for 5:3 (*p* = 0.083). In the surrogate data, the distribution patterns were not clear and no statistical significance was found, with only a trend for 7:4 (*p* = 0.093).

### Effect of maternal respiratory rate

The median maternal respiratory rates found in the subjects’ 18 min. data sets ranged from 12.8 to 20.7 cpm (mean ± SD: 17.0±1.9 cpm). Although the rates were somewhat higher in the Control group, this difference was not statistically significant (Control vs. Exercise: 17.4±1.8 cpm vs. 16.6±1.9 cpm, *p* = 0.196). Comparing the respiratory rates of the subjects’ SE gave similar results at somewhat slower rates: 16.3±2.1 vs. 15.6±2.1, *p* = 0.376.

**Figure 4:**
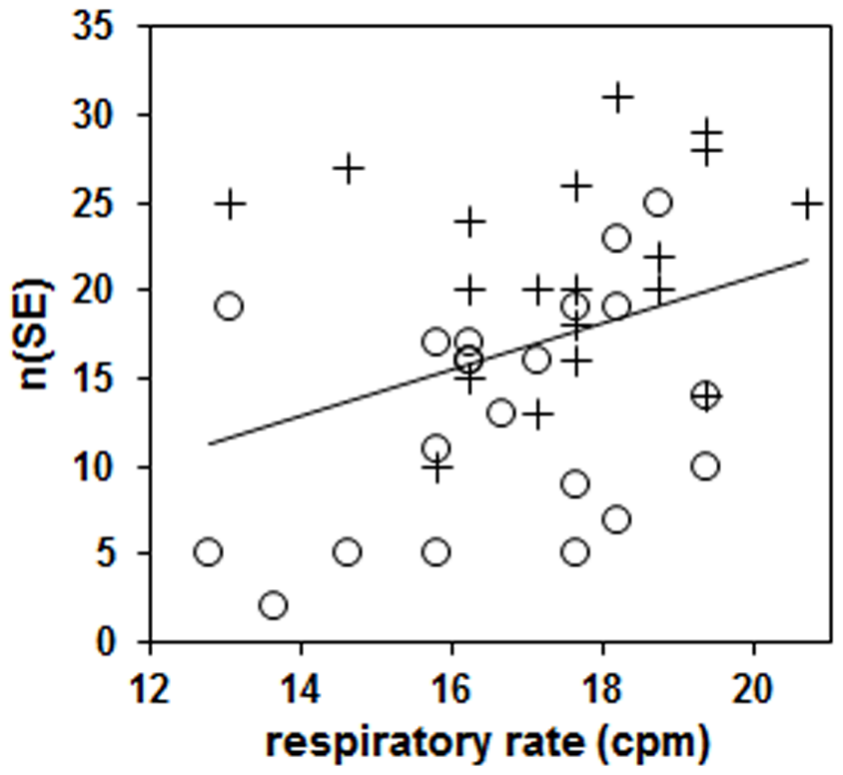
Relationship between the median maternal respiratory rate and the number of synchronization epochs (SE) found per data set. Data points are shown separately for the Control (+) and Exercise (O) data, the regression line shown is calculated over all data points.

There was a significant relationship between the number of SE found per data set and the respiratory rate: n(SE) = 1.3 * respiratory rate –5.4, *p*_slope_ = 0.034 (see also Fig. 4). Separating the subjects into groups with respiratory rates above and below 18 cpm [14] showed that those with slower rates had significantly fewer SE (15.1±6.9 vs. 20.5±7.5, *p*<0.001). Examining the Control and Exercise groups separately gave similar results (Control: 19.5±5.4 vs. 24.1±5.9, *p*<0.001 and Exercise: 11.7±6.0 vs. 16.3±7.2, *p*<0.001), whereby the Exercise subgroup values also tended to be lower than the corresponding Control values.

We also found a dependency of the total SE duration per data set on respiratory rate (total duration(SE) = 26 * respiratory rate –153, *p*_slope_ = 0.040) and examination of the subgroups showed results similar to the number of SE but these results were not statistically significant.

## Discussion

Our aim was to determine if differences in maternal and fetal heart rate and HRV resulting from maternal exercise would be associated with differences in maternal-fetal heart rate synchronization characteristics between the two groups. The main finding of this study is that epochs of synchronization of fetal and maternal heart rates occurred less often in pregnancies in which the mothers had exercised regularly. In the Control subjects, on average 21 SE were found in the 18 min. of heart rate data, in the Exercise group this was only 13 SE. The subject groups also differed with respect to two main aspects: their overall HRV and the maternal respiratory rates. With respect to HRV, the Control mothers and fetuses had a lower combined variance, as quantified by *Δq*, due to the lower HRV in the fetuses and, to a lesser extent, in the mothers. This corresponds to similar results previously reported [12]. The relationship between HRV and the number of SE found also shows a clear tendency to more SE at lower HRV (see Table 3 and Fig. 2). The fact that the occurrence of SE is less likely when HRV is high may be explained in particular by the greater beat-to-beat differences. In our previous work, it could be shown that the occurrence of SE was significantly lower in data sets with high RMSSD values [14]. This suggests that greater differences in the duration of successive maternal heart beats reduce the likelihood of fetal-maternal heart rate coupling. This will be so because fetal short-term beat-to-beat variability, although also increased, will be appreciably lower than the maternal short-term variability, making the adaptation of the fetal system to large changes in maternal beat duration difficult. The fact that Exercise group had appreciably higher maternal beat-to-beat differences may thus have hindered the occurrence of SE.

A second factor that has been shown to be associated with increased occurrence of fetal-maternal heart rate synchronization is the rate of maternal breathing. We have demonstrated that fast respiratory rates (20 cpm) were more conducive to synchronization whereas slow rates (10 cpm) led to fewer SE [14] [15]. Although maternal breathing was not controlled in the present study, we did find a significant relationship between maternal estimated spontaneous respiratory rate and the number of SE in each data set, with more epochs present at higher rates. Subjects with respiratory rates above 18 cpm had significantly more SE, in agreement with the work mentioned above. Looking at the two groups separately gave similar results, i.e. both the Control and Exercise groups with respiratory rates above 18 cpm had more SE than the respective subjects with rates below 18 cpm. The respiratory rates of the Control group were not significantly higher but there were relatively more Control subjects with rates above 18 cpm (37% Control vs. 29% Exercise, see also Fig. 4). This implies that respiratory rate also led to more SE being identified in the Control group. One might expect that the increase in SE at higher respiratory rates is due to a concomitant lower HRV, i.e. that lower HRV and not so much fast breathing induces the occurrence of synchronization. However, we found only a weak relationship between overall combined variance and respiratory rate (*r* ^2^ = 0.08), indicating that less than 10% of the variance found in *Δq* could be explained by the respiratory rate.

The results indicate that fetal-maternal heart rate coupling is generally weak. Creating favorable conditions for synchronization seems to depend on both HRV and respiratory rate as they influence momentary changes in both fetal and maternal heart rate. The mothers who exercised had slower breathing rates and had a higher combined fetal-maternal HRV which seem to have hindered the heart rate synchronization. Examination of the characteristics of the SE in the two groups supports this. The *n*:*m* combinations of the SE found in sedentary mothers were more concentrated in the mid-range (4:3, 3:2, 5:3, 7:4) whereas in the Exercise group the distribution of the *n*:*m* combinations was broader with relatively more SE at the extremes (e.g. 5:4 and 9:4/7:3) than in the sedentary group. This was due to different heart rates, i.e. in the Exercise data the range of the heart rates, in particular the maternal, was larger which led to the coincidence of fetal and maternal heart rates both close to each other as well as quite far apart. In both subject groups, the HRV in the SE was dramatically lower than the HRV in their complete 18 min. data sets. This can be explained by the comparative shortness of the SE duration as well as the need for a degree of heart rate stability in order to maintain the synchronization. Nonetheless, within the SE the Exercise group tended to somewhat higher HRV values. The basic overall differences we found between the two groups in their complete data sets are thus reflected in the characteristics of the SE, albeit with substantially lower HRV. This would support the suggestion that the heart rates and respiratory rates play a central role in the enabling of synchronization.

Using surrogate data analysis, we also addressed the question whether the SE we found represent true physiological interaction between mother and child or whether they were the result of chance coordination between the heart rates. We used the method of twin surrogates to reorder the maternal RR intervals and uncouple their temporal relationship to the fetal RR intervals. This method has been shown to adequately reproduce the linear and nonlinear characteristics of the system under examination [20]. After analyzing the surrogate data in the same manner as the original data and comparing the results, we found no differences in the overall HRV measures. However, there were also no differences with respect to the number of SE found and their characteristics (e.g. their HRV, the Control/Exercise proportions as well as their distribution over the *n*:*m* combinations). This would seem to suggest that the SE found in the original data may simply result from chance coincidence of the fetal and maternal heart rates to produce short-term stable *n*:*m* combinations. On the other hand, we found two interesting differences between the original and surrogate SE. Firstly, the surrogate SE were slightly but significantly shorter than the original SE. Although the difference is small and the *p*-value is close to the level of significance, we consider this result as being non-trivial for the following reason. The similarity of the results for original and surrogate data mentioned above suggests that a substantial proportion (but not necessarily all) of the SE found in the original data were probably spurious and will have had durations similar to the surrogate SE. Thus the difference which was indeed found in the duration, even if small in absolute terms and with a *p*-value close to the level of significance, may be considered meaningful. Furthermore, we also examined the phase timing of the fetal beats with respect to the maternal beats in those *n*:*m* combinations with a reasonable number of SE. In the surrogate data we found no significant deviations from a uniform distribution of the fetal beat timings. However, in the original data, a phase preference was apparent, suggesting a physiological link between the fetal and maternal cardiac oscillators. These results correspond to previous work in which we report similar evidence for a phase preference in the original data [13] [14].

On the basis of the results described here as well those in previous reports, we assume that some proportion of the SE found in the original data are due physiological interaction between the mother’s heart rate and that of her child. Evidence for fetal-maternal heart rate synchronization has been reported using other approaches [24] [25] [26]. Whereas these studies did not control for possible factors involved in synchronization, our previous work indicated that the rate of maternal respiration influences the occurrence of epochs of synchronization [14]. The present study supports this notion of maternal influence and further suggests that maternal aerobic exercise also plays a (damping) role in enabling synchronization. These results imply that the maternal condition is an influencing factor. Data driven model-based analysis has indeed identified a consistent influence of the duration of maternal heart beats preceding the fetal beats during epochs of synchronization [27]. This influence could not be observed in beat trains without synchronization.

If the maternal condition predisposes the fetal cardiac system to couple with the maternal system, the question arises as to whether synchronization is a marker for physiological health or development. In infancy, the concept of “synchrony” has been described as parent-infant behavioral coordination which develops postnatally and into infanthood. This interaction between infant and caregiver (dyadic synchrony) can be observed in terms of coordination of body orientation, body movement, facial expression, vocalization and visual gaze [28]. Furthermore, studies examining the behavioral interaction between mothers and their three-month old infants have shown that during episodes of dyadic synchrony an increase in correlation between their heart rhythms may be observed [29] [30]. Thus, cardiac interaction may be seen as an aspect of behavioral interaction. If early human development is seen as a continuum from the antenatal to the postnatal stage [31], then fetal-maternal heart rate synchronization may well reflect the capacity of the fetus to engage with its environment. What our results indicate is that the propensity for cardiac activity to synchronize in the antenatal condition will be modulated by factors influencing heart beat duration. The fact that pregnancies in which the mothers regularly engaged in aerobic exercise were associated a lower rate of synchronization occurrence suggests that, although the conditions were unfavorable, synchronization nonetheless occurred.

The mechanism by which heart rate synchronization occurs remains unclear. We have suggested that the coupling is mediated by the fetal auditory system which is capable of detecting maternal heart beats [32] and that the perception of the beats represents the weak forcing signal permitting the system to couple [33]. One way to test this hypothesis would be to design a study in the immediate postnatal period in which various possible influencing factors could be examined under controlled conditions. The results of such studies might then offer the possibility to actively induce or hinder synchronization and to aid in the assessment of the physiological role heart rate synchronization between mother and child plays in early human development.

## Supporting Information

Data S1 **Excel file containing the data for each fetal-maternal pair.** Each pair is either in the Control or Exercise group. For each pair, the timing of the fetal and maternal R times as well as the maternal signal strength at each R peak are given.
(XLSX)
